# Spousal discordance on fertility preference and its effect on contraceptive practice among married couples in Jimma zone, Ethiopia

**DOI:** 10.1186/1742-4755-11-27

**Published:** 2014-04-04

**Authors:** Tizta Tilahun, Gily Coene, Marleen Temmerman, Olivier Degomme

**Affiliations:** 1 College of Public Health and Medical Sciences, Jimma University, Jimma, Ethiopia; 2 Rhea, Research Centre on Gender and Diversity, Brussels University, Brussels, Belgium; 3 International Centre for Reproductive Health, Department of Obstetrics and Gynecology, Ghent University, Ghent, Belgium

**Keywords:** Fertility preference, Couples, Contraception, Spousal discordance

## Abstract

**Objectives:**

To assess spousal agreement levels regarding fertility preference and spousal communication, and to look at how it affects contraceptive use by couples.

**Methods:**

We conducted a cross-sectional study to collect quantitative data from March to May 2010 in Jimma zone, Ethiopia, using a multistage sampling design covering six districts. In each of the 811 couples included in the survey, both spouses were interviewed. Concordance between the husband and wife was assessed using different statistics and tests including concordance rates, ANOVA, Cohen’s Κ and McNemar’s test for paired samples. Multivariate analysis was computed to ascertain factors associated with contraceptive use.

**Results:**

Over half of the couples wanted more children and 27.8% of the spouses differed about the desire for more children. In terms of sex preference, there was a 48.7% discord in couples who wanted to have more children. At large, spousal concordance on the importance of family planning was positive. However, it was the husband’s favourable attitude towards family planning that determined a couple’s use of contraception. Overall, contraceptive prevalence was 42.9%. Among the groups with the highest level of contraceptive users, were couples where the husband does not want any more children. Spousal communication about the decision to use contraception showed a positive association with a couple’s contraceptive prevalence.

**Conclusions:**

Family planning programs aiming to increase contraceptive uptake could benefit from findings on spousal agreement regarding fertility desire, because the characteristics of each spouse influences the couple’s fertility level. Disparities between husband and wife about the desire for more children sustain the need for male consideration while analysing the unmet need for contraception. Moreover, men play a significant role in the decision making concerning contraceptive use. Accordingly, involving men in family planning programs could increase a couple’s contraceptive practice in the future.

## Introduction

As we are approaching the year 2015, governments across the world are evaluating the progress that was made over the last decade in meeting the Millennium Development Goals (MDG). Of the eight goals, MDG5 “Improve maternal health” has made one of the slowest progresses, and it seems unlikely that the target of reducing the 1990 maternal mortality ratio by three quarters by 2015 will be reached. Sub-Saharan Africa as a whole has the world’s highest maternal mortality ratio, a contraceptive prevalence of only 25 per cent, and low levels of skilled attendance at birth [1].

In developing countries, research indicated that the number of women wanting to avoid pregnancy and therefore needing effective contraception, increased substantially, from 867 million (57%) to 1520 million in 2012 [2]. However, the unmet need for modern contraceptives was still very high, particularly in sub-Saharan Africa, south Asia, and western Asia [2]. Although it remains a real challenge to ensure consistent access to family planning services, evidence from Demographic and Health Survey (DHS) data shows that it is women and men’s attitude towards contraception that is a more important barrier to overcome [3].

From a medical perspective, most contraceptive methods are developed for women, and family planning services have primarily been provided to individual women too, not to couples. As a consequence, men have been virtually ignored by family planning programs for several decades [4]. However, it is becoming increasingly clear that husbands do play a crucial role in fertility decision making throughout the world. Research in rural India exploring concordance between husbands and wives regarding reproductive intentions and contraception, reported that within the surveyed community, no case was found of a husband reporting an unmet need, although several wives did experience it [5].

Similarly, findings from Kenya show that contraception is much more likely to be used when the husband rather than the wife wants to cease childbearing [6]. Moreover, spousal communication has a strong association with contraceptive use. Indeed, findings of Lasee et al. [7] show that women’s use of contraceptives is additionally determined by their husbands approval [7].

The above mentioned literature stresses the need to include both husbands and wives when assessing family planning attitudes. Preventing unintended pregnancies is thus not merely the responsibility of women; yet, the relationship between men’s attitude and the unmet need for family planning are considerably understudied, as evidenced by the paucity of scientific articles on the subject indexed in Pubmed during the last 10 years. Even though there were studies conducted about family planning and men’s attitude, very few dealt with male involvement. This is an important gap since, contrarily to surveys pertaining to reproductive health events where the wife’s response can be taken as a proxy for the couple’s response, in studies assessing family planning attitudes and intentions, there is a need to collect unbiased information from husbands and wives separately.

In Ethiopia, contraceptive use among married women has increased considerably during the last decade, going from 15% in 2005 to 29% in 2011 [3,8]. Within the country’s largest region, Oromiya, contraceptive prevalence rate (CPR) levels of 26.2% were reported in 2011 [8]. Over the same period, the total fertility rate (TFR) at national level decreased from 5.4 to 4.8 children per woman [3,8]. Still, in Ethiopia 25% of currently married women has an unmet need for family planning [8]. At the regional level, the total unmet need for family planning ranges from 30% in Oromiya to 11% in Addis Ababa [8,9]. Factors that have been identified as causes for this situation include religious prohibition and insufficient knowledge of contraception, but also the husbands’ opposition [10] and a lack of communication between the spouses [11].

It is well recognized that agreement on contraceptive use between spouses is one of the reasons mentioned for decreasing the unmet need for family planning [5]. This may be explained by the determinant effect of having the approval of family planning and contraceptive use by the spouse [10]. As such, to explore the concordance and discordance between husband and wife could mean a valuable contribution to current research, since it affects a couple’s contraceptive use. In this paper, we assessed spousal concordance levels regarding partner’s fertility preference and spousal communication and how it affects contraceptive use. The second objective of this study is to examine concordance between spouses on reporting the male involvement in family planning. This could contribute to identify not only how husband-wife agreement affects contraceptive use, but also to forward ways to decrease the unmet need for family planning.

## Methods

The study was conducted in Jimma Zone, one of the 14 administrative zones of the Oromyia region in south-western Ethiopia. Its capital, Jimma, is situated 352 km to the south west of the national capital Addis Ababa. Jimma Zone has 17 *woredas* (districts) and one special zone. According to the 2007 national census, its total population is approximately 2,486,155 with an area of almost 15,568.58 km^2 ^[12]. The rural part accounts for 89.5% of the total population size of the zone, in which the dominant ethnic group is the Oromo. The study was mainly conducted in these rural parts.

We conducted a cross-sectional study in Jimma Zone from March to May 2010, using quantitative data collection techniques. Sampling was done using a multi-stage design with three districts (locally *woredas*) as primary sampling units (PSU), and six localities (locally *kebeles*) as secondary sampling units (SSU). The study covered six localities that were randomly selected from within three districts. Census was done and 1507 respondents were interviewed in six *kebeles* of the study area. Minitab version 14 statistical software was used to determine the sample size, we assumed 80% power, a baseline contraceptive prevalence of 50%, to detect 10% variation in the intervention arm, and 95% confidence level. This resulted in a minimum sample size of 388. Adding a 10% contingency, 427 married couples for the intervention and 427 married couples for the control group; in total 854 married couples were included in the study. These households were randomly sampled among the six selected localities based on a computer generated random number until the required size was achieved. Two paired samples – one for men and one for women (husbands and their wives) – were selected. Inclusion criteria for the women that were interviewed, were reproductive age (15–49 years) and not being pregnant. The men included in the study were those who were married to the selected women. Polygamous couples were not included in the study.

We used semi-structured questionnaires with different questions for male and female respondents, yet containing similar concepts. Data was collected on basic background characteristics, reproduction and fertility preference (questions such as Do you want to have additional children? If yes do you want a boy or a girl?), knowledge on family planning (including types of contraceptives, advantage and side effects of contraceptives), contraceptive practice (questions like Have you ever used anything or tried in any way to delay or avoid having child?), husband-wife communication on family planning, and reasons for not using contraceptives. Male in family planning was defined as a husband who uses contraceptives or as a husband who has intention to support the contraceptive use by his wife, for instance a husband who has the intention to participate in the visits to family planning services, who has the intention to finance and purchase contraceptives, or who was actually present on the day his wife had an appointment at the clinic or at family planning services. Discussion about family planning between husband and wife was defined as “ever had discussion with your spouse about using family planning methods in the last 3 months prior to this study survey.” Questionnaire items were adapted and modified from instruments used in the 2005 Ethiopian DHS [3].

Pre-test of the questionnaires was done among 5% of the total sample with couples in the area other than the study site, but with a similar set-up. The purpose of pre-testing is to ascertain problems with the data collection tool and to find possible solutions. Six male and six female data collectors and three supervisors participated in the data collection. The participants were included based on the following criteria: resident in the community for more than six months, being married, and women aged between 15–49.

Collected data was checked for consistency, coded and then entered into the computer using Epi-data. After editing, the data analysis was done via STATA® 10 for Windows® and R version 2.15.11.

Concordance between husband and wife was assessed using different statistics and tests including concordance rates, Cohen’s κ and McNemar’s test for paired samples. A commonly accepted interpretation of the level of agreement for Cohen’s κ is: poor agreement (κ < 0.2), fair agreement (0.2 < κ < 0.40), moderate agreement (0.40 < κ < 0.60), good agreement (0.60 < κ < 0.80) and very good agreement (0.80 < κ < 1.00) [13]. Since the value of Cohen’s κ can be highly influenced by the prevalence of the observed phenomenon, we also calculated a prevalence-and-bias-adjusted (κ PABAK) that corrects for differences in prevalence and is considered a better statistic if the prevalence is low [14,15].

We calculated unadjusted odds ratios for current contraceptive use in function of selected variables of the husbands and the wives. We did so for 4 distinct groups based on the spouses’ desire for more children, i.e. 1) couples in which both spouses desire no more children; 2) couples in which the wife does not want additional children, but the husband does; 3) couples in which the husband does not want additional children, but the wife does; and 4) couples in which both spouses desire more children. Moreover, ANalysis of VAriance between groups (ANOVA) was applied to determine factors associated with current contraceptive use among different the groups. Finally, multivariate analysis was carried out to identify possible correlation between factors associated with contraceptive use and P-value less than 0.005 was considered as level for significance.

Ethical clearance for this study was obtained from the research and ethics committee of the College of Public Health and Medical Sciences at Jimma University, Southwest Ethiopia and Ghent University Ethical Committee, Belgium. Consent was informed to the survey respondents and written consent was taken.

## Results

### Respondents’ characteristics

We obtained data from 811 couples corresponding to a response rate of nearly 95%. The vast majority of couples consisted of farmer husbands and farmer wives, whereas nearly eight per cent of the couples were farmer husbands and housewives. Among the couples, the predominant religion was Islam, and only in 18 (2.2%) couples, husband and wife belonged to different religious groups. In terms of education, 159 (19.6%) couples reported not being able to read nor write, in 373 (46.0%) couples, the wife was illiterate while the husband had received education, and in 148 (18.2%) couples, both spouses attended primary education.

Husbands were on average 8.3 years older than their wives, even though wives were older than their husbands in 58 (7.2%) couples. Similarly, men are 5.8 years older when they marry the very first time than women who marry the very first time. Women had more children than their husbands in 178 (21.9%) couples, less than their husbands in 111 (13.7%) couples and the same number in 522 (64.4%); overall, women had on average 0.17 children more than their husbands (p = 0.002) (see Table 1).

**Table 1:** Selected demographic characteristics of 811 couples, Jimma zone, Ethiopia, 2014

	**Husbands**	**Wives**	**Difference**	***P-value***
**Mean age (years)**	38.5 (37.7; 39.4)	30.3 (29.6; 30.9)	8.3 (7.6; 8.9)	*<0.001*
**Mean age at first marriage (years)**	23.5 (22.9; 24.2)	17.7 (17.1; 18.4)	5.8 (4.9; 6.6)	*<0.001*
**Mean number of children from all marriages**	3.2 (3.1; 3.4)	3.4 (3.3; 3.6)	-0.17 (-0.28; -0.07)	*0.002*
**Mean ideal number of children before starting family planning**	4.3 (4.1; 4.5)	4.6 (4.5; 4.7)	-0.37 (-0.15; -0.59)	*<0.001*

### Fertility preferences

On average, women envisaged to start limiting the number of pregnancies through family planning after bringing forth 4.6 children. This was 0.37 children more than their husbands (p < 0.001).

Table 2 shows the levels of concordance for indicators of fertility preference. Overall, there is a slight to fair agreement between the spouses with Cohen’s κ values between -0.057 and 0.374, but prevalence-and-bias-adjusted κ (PABAK) values between 0.189 and 0.445 (see Table 2). Among the couples that disagreed with respect to the desire for having additional children, twice as many couples consisted of a husband wanting an additional child compared to those where it was the wife desiring another child (150 (18.5%) vs 75 (9.2%)).

**Table 2:** Spousal agreement on fertility preference and their perceptions on family planning importance among couples in Jimma zone, Ethiopia, 2014

	**Concordance**	**Discordance**	**Kappa**		**P-PABAK**	
**Desire for more children (N = 811)**	586 (72.2%)	225 (27.8%)	0.374		0.445	
**Sex preference (N = 429)**	224 (52.2%)	209 (48.7%)	0.183		0.189	
**Had ever received information on abortion (N = 809)**	564 (69.3%)	245 (30.7%)	-0.057		0.394	
**Discussed family planning in the couple in the 3 months prior to the survey (N = 394)**	273 (69.3%)	121 (30.7%)	0.212		0.373	
**FP is very important for…**	**Husband (%)**	**Wife(%)**	**Spouses agree it is very important (%)**	**Spouses agree it is not very important (%)**	**Kappa**	**PABAK**
**…delaying the first child.**	73.2	52.5	39.8	14.1	0.055	0.078
**…spacing births.**	81.4	62.9	54.4	10.1	0.153	0.290
**…limiting the number of children.**	77.7	62.9	52.7	12.1	0.177	0.295
**…the benefit of family members.**	71.3	56.2	44.3	16.8	0.177	0.221
**…economic reasons.**	79.4	67.0	54.9	8.5	0.085	0.268
**…the mother’s health.**	87.7	72.1	62.6	2.8	0.036	0.309
**…children’s health.**	88.2	74.8	65.6	2.4	0.038	0.358

PABAK = Prevalence And Biased Adjusted Kappa.

We asked all spouses if family planning had been discussed previously within the couple. In 211 (26%) cases, we obtained different answers from husband and wife (Cohen’s κ = 0.45; PABAK = 0.48). In 145 (17.9%) couples, the husband answered positively while the wife did not, and in 66 (8.1%) it was the opposite. In the 394 (48.7%) couples where previous discussion was reported by both spouses, 273 (69.3%) agreed that family planning had been discussed in the course of the last 3 months. Among this group, the concordance with respect to the desire for more children was not higher than in the rest of the sample (Concordance rate: 72.1% vs 70.0%; Cohen’s κ = 0.307 vs 0.322).

The variable that showed the lowest level of agreement was with respect to preference for the sex of the next child. Out of the 429 couples where both spouses wanted more children, there were 139 (32.4%) who showed no preference at all; in 61 (14%) couples, both spouses desired a boy, while in 20 (4.6%) couples, they both desired a girl. In 183 (42.7%) couples, discordance existed, since one of the spouses had a preference for one particular sex while the other spouse preferred another. True discordance was found in 26 (6%) couples of which 22 (5.1%) consisted of the husband desiring a boy and the wife desiring a girl. Among the 150 husbands who wanted more children while their wives did not, 65 (43.3%) wanted an additional boy and 17 (11.3%) a girl; among the 75 women wanting more children in spite of their husband, 16 (21.3%) had a preference for a boy and 7 (9.3%) for a girl. These findings suggest that the desire – in particular the husband’s desire - for another son is a critical element resulting in discordance within the couple, both with respect to having another child as to the sex of the next child.

Most of the couples agreed that family planning was a good thing to limit pregnancies; 52.7% (427) of the couples were unanimously in favour of it (see Table 2). The degree to which husbands and wives were able to correctly evaluate their spouse’s attitude towards family planning, was 82% (665) among women and 79.3% (643) among men. We observed higher levels of correctly evaluating the spouse’s attitude in couples that reported having discussed family planning in the past compared to those that had not (among women: 89.6% vs 76% (χ^2^ (1, N = 780) = 24.007, p < 0.001); among men: 89.9% vs 75.1% (χ^2^ (1, N = 754) = 27.323, p < 0.001)).

Family planning was seen by most of the husbands as very important, with proportions ranging from 71.3% to 88.2% (see Table 2). In most cases, importance to family planning was given in common by both spouses for the purpose of preserving the mother’s and the children’s health. These motives showed the highest level of concordance, with 62.6% for the mother’s and 65.6% for the children’s health (see Table 2). However, there was prioritization alteration in spousal report about the benefits of family planning; husbands tend to say family planning is important for demographic and health reasons whereas women put forward economic explanations. On top of that, limiting the number of children was given as an advantage of family planning by men, while women chose family planning for spacing purposes.

An additional factor significantly associated with spousal discordance is a previous unintended pregnancy (χ^2^ (3, N = 811) =22.376, p = ≤0.001); Cohen’s κ was 0.41 (PABAK = 0.47) for couples that had never faced an unintended pregnancy (N = 687) with 505 (73.5%) couples concurring, as opposed to 0.15 (PABAK = 0.31) among couples that had (N = 124; with 81 (65.3%) concurring). In 37 of the latter couples (30%), it was the husband who wanted more children when the wife did not, while in 6 couples (4.8%), the opposite was true.

The level of discordance was positively associated with an increasing age of the wife (χ^2^ (6, N = 806) = 33.103, p < 0.001) and to a lesser extent with an increasing age of the husband (χ^2^ (11, N = 808) = 19.895, p = 0.047), as well as with higher numbers of children the woman (χ^2^ (7, N = 811) = 35.247, p < 0.001) and the husband had (χ^2^ (7, N = 811) = 42.166, p < 0.001). These findings suggest that discordance is of bigger importance in older couples than in younger ones where fertility preferences seem to concur. 

### Family planning

In 748 (92.2%) couples, both spouses had heard of ways or methods they could use to limit pregnancies; in another 47 (5.8%) couples, only the woman had ever heard of it, while in 12 (1.5%) couples, only the man had. On average, no difference was found between the number of contraceptive methods the husband and wife knew (Wilcoxon’s W = 133764, p = 0.072); in 107 (13.2%) couples, the wife knew as many methods as the husband, in 326 (40.2%) she knew more and in 378 (46.6%) she knew less. The better knowledge of husbands compared to their wives was negatively correlated to increasing age (p < 0.001), meaning that in younger couples, the husband knows more methods than the wife.

Contrary to the knowledge of methods, we noticed a difference between spouses in knowledge of the use of contraceptives, with women having a better knowledge than their husbands (McNemar’s χ^2^ (1, N = 756) = 81.679, p = ≤0.001). Of the 748 couples that had heard of family planning methods before, 389 (45.1%) consisted of both spouses knowing how to use contraceptives, in 261 (34.9%), the husband did not know how to use them, in 90 (12%) the wife did not. Finally, 14 (1.7%) couples did not know if a woman could get pregnant or not while using contraceptives.

Out of 811 couples, 350 (43.1%) reported that they were currently using a method of family planning; in 348 (42.9%) of these, it was only the woman who was using a method, while in 2 (0.2%) couples, it was both the husband and the wife (see Table 3). Among the 461 couples in which no type of family planning was currently used, 96 (20.8%) couples concurred that they did not desire another child, while in 122 (26.5%) couples, it was only one of the two spouses who did not. There were 451 (97.8%) and 442 (95.9%) couples that agreed that they were not deterred by the cost nor by the lack of knowledge of where to get access to family planning services, respectively. On the other hand, husbands in 148 (32.1%) couples mentioned that the lack of knowledge of the most appropriate method was the reason for not using any method at all, even though their wives did not mention this reason, and 90 (19.5%) women said that fear of side effects contributed to not using any type of contraception, while their husbands did not express this fear. These two reasons were more frequently reported by older men (defined as equal or older than the male sample’s mean age of 39) (κ^2^(1, N = 806) = 3.681, p = 0.055) and older women (defined as equal or older than the female sample’s mean age of 30) (χ^2^ (1, N = 459) = 4.656, p = 0.031) respectively.

**Table 3:** Univarate analysis of age, attitude towards family planning and unadjusted odds ratios for current contraceptive use, Jimma zone, Ethiopia, 2014

	**Total**	**Both spouses don’t desire more children (group**_**1**_**)**	**Woman doesn’t desire more children (group**_**2**_**)**	**Man doesn’t desire more children (group**_**3**_**)**	**Both spouses desire more children (group**_**4**_**)**
N	811	151	150	75	435
Median age wife (yrs)	30	38	32	30	25
Median age husband (yrs)	36	45	40	38	32
Current contraceptive use	42.9% [39.5;46.3]	36.4% [28.8;44.6]	38.7% [30.8;47]	60.0% [48;71.2]	44.1% [39.4;48.9]
**Attitude towards FP**					
*Husband is favourable to his wife using a FP method*					
Reported by the husband	6.24*** [3.60;11.61]	9.84*** [2.27;88.36]	3.29 [0.85;18.57]	11.00* [1.19;515.7]	5.96*** [2.7;14.9]
Reported by the wife	2.60*** [1.95;3.49]	3.81*** [1.77;8.22]	2.25* [1.09;4.65]	6.00** [1.91;19.26]	2.46*** [1.64;3.71]

*P < 0.05, **P < 0.01, ***P < 0.001.

Husbands and wives were asked who in the couple had the final decision on using family planning methods. In 573 (71.1%) out of 805 couples, both spouses concurred that the final decision should be made by both of them. In 129 (16.0%) couples, however, the wife stated the final decision is with the husband while the husband answered it was a shared decision; we observed the opposite in 44 (5.5%) couples. We assessed male involvement using three questions: intention to go to family planning services, intention to pay for contraception, and actual presence during family planning consultation with the wife. In the 350 (43.1%) couples where a method of family planning was currently used, 99 (28.3%) spouses reported that the husband had the intention to participate in a consultation; in 58 (16.6%) couples, the husband stated he intended to participate in such a consultation, while the wife said he was not, and in 91 (26%), the wife said he would participate, while he said the opposite.

### Factors associated with current use of contraception

Table 4 shows the odds ratios for current use of any method of contraception in four distinct groups: 1) couples in which both spouses do not desire more children (group_1_; N = 151); 2) couples in which the wife does not want additional children, but the husband does (group_2_; N = 150); 3) couples in which the husband does not want additional children, but the wife does (group_3_; N = 75); and 4) couples in which both spouses desire more children (group_4_; N = 435). The group in which the husband was the only one not desiring more children had a significantly higher level of current contraception use than the other groups (see Table 4).

**Table 4:** Univarate analysis of perception of spouses on family planning importance, male involvement and unadjusted odds ratios for current contraceptive use, Jimma zone, Ethiopia, 2014

	**Total**	**Both spouses don’t desire more children (group**_**1**_**)**	**Woman doesn’t desire more children (group**_**2**_**)**	**Man doesn’t desire more children (group**_**3**_**)**	**Both spouses desire more children (group**_**4**_**)**
**Perception of the husband**					
*Husband considers FP is very important for…*					
…Delaying the first child.	1.65	2.00	0.42	2.05	2.46***
	[1.20;2.29]	[0.87;4.86]	[0.2;0.87]	[0.4;11.25]	[1.5;4.07]
…Spacing births.	1.72**	6.38***	0.47	0.89	2.05*
	[1.19;2.52]	[2.05;26.15]	[0.2;1.09]	[0.13;5.02]	[1.16;3.7]
…Limiting the number of children.	1.81***	3.37**	0.78	1.6	1.96*
	[1.28;2.58]	[1.36;9.15]	[0.36;1.74]	[0.33;7.68]	[1.16;3.37]
…The benefit of family members	1.16	2.06	0.49	0.5	1.48
	[0.85;1.58]	[0.97;4.5]	[0.22;1.09]	[0.15;1.56]	[0.92;2.38]
…Economic reasons.	1.40	3.07**	0.33*	0.5	2.4**
	[0.99;1.99]	[1.28;7.93]	[0.13;0.83]	[0.14;1.64]	[1.34;4.41]
…The mother’s health.	1.70*	2.97	0.33*	0.73	3.17**
	[1.10;2.68]	[1;10.65]	[0.11;0.94]	[0.06;5.54]	[1.48;7.34]
…Children’s health.	1.73*	4.31**	0.24**	1.59	2.59*
	[1.11;2.77]	[1.35;17.98]	[0.07;0.73]	[0.11;23.04]	[1.23;5.83]
**Perception of the wife**					
*Wife considers FP is very important for…*					
…Delaying the first child.	2.24***	2.74**	3.59**	1.49	2.19***
	[1.68;2.98]	[1.31;5.74]	[1.57;8.6]	[0.53;4.21]	[1.46;3.29]
…Spacing births.	3.22***	2.23*	3.43**	3.06*	3.7***
	[2.36;4.41]	[1.06;4.76]	[1.5;8.24]	[1;9.46]	[2.36;5.87]
…Limiting the number of children.	2.71***	3**	1.93	4.75**	2.63***
	[2.00;3.68]	[1.41;6.54]	[0.9;4.22]	[1.44;16.41]	[1.7;4.08]
…The benefit of family members	2.60***	2.54*	2.21*	4.58**	2.53***
	[1.94;3.49]	[1.22;5.3]	[1.03;4.81]	[1.5;14.2]	[1.67;3.85]
…Economic reasons.	3.31***	3.82***	3.5**	4.15*	2.98***
	[2.40;4.60]	[1.73;8.71]	[1.46;9.04]	[1.25;14.4]	[1.88;4.77]
…The mother’s health.	3.03***	3.11**	3.99**	3.06	2.96***
	[2.16;4.29]	[1.36;7.46]	[1.47;12.56]	[0.97;9.76]	[1.81;4.9]
…Children’s health.	3.41***	6.35***	6.21***	1.59	3.2***
	[2.39;4.96]	[2.38;19.67]	[1.98;25.6]	[0.52;4.81]	[1.89;5.56]
**Involvement of the husband**					
*Husband makes plans…*					
Intention to go jointly to get family planning delivery.	4.17***	6.17***	1.62	8.5***	4.67***
	[3.07;5.70]	[2.81;13.66]	[0.78;3.36]	[2.46;31.3]	[2.94;7.47]
Intention to finance and purchase contraception.	5.13***	7.09***	2.07	10.86***	6.21***
	[3.76;7.05]	[3.17;15.98]	[1.00;4.34]	[3.19;38.5]	[3.83;10.23]
Actual presence during family planning consultation.	1.46**	2.2	1.83	0.59	1.55*
	[1.10;1.94]	[0.89;5.42]	[0.89;3.75]	[0.17;2.12]	[1.04;2.3]
**Practices within the couple**					
*FP was previously discussed within the couple*					
Reported by the husband	5.62***	5.85***	3.01**	14***	5.98***
	[3.98;8.07]	[2.55;14]	[1.31;7.23]	[3.19;82.42]	[3.57;10.27]
Reported by the wife	12.55***	20.87***	11.56***	21***	10.21***
	[8.79;18.25]	[7.57;64.95]	[4.82;28.99]	[4.74;122.9]	[6.17;17.17]
*Wife can use FP method without husband's consent*					
Reported by the husband	0.97	1.38	0.85	0.74	0.97
	[0.70;1.35]	[0.6;3.11]	[0.37;1.93]	[0.19;3.01]	[0.61;1.53]
Reported by the wife	0.75	1.01	1.21	0.85	1.05
	[0.56;1.01]	[0.48;2.07]	[0.59;2.47]	[0.27;2.72]	[0.69;1.61]
*Husband is responsible for final decision-making regarding FP*					
Reported by the husband	0.41**	0.32	0.49	0*	0.58
	[0.23;0.73]	[0.03;1.62]	[0.11;1.75]	[0;0.67]	[0.24;1.33]
Reported by the wife	0.81	1.15	1.08	0.36	0.74
	[ 0.56;1.17]	[0.4;3.1]	[0.46;2.48]	[0.05;2.05]	[0.44;1.25]

*P < 0.05,**P < 0.01,***P < 0.001.

A positive attitude of the husband towards family planning was consistently associated with a higher contraceptive use. Furthermore, ANOVA analysis showed that this factor explained a larger share of the variance in the two groups where the husband did not desire more children (group_1_ = 17%; group_3_ = 13% vs. group_2_ = 2%; group_4_ = 8%) (Table 4).

In group_1_ and group_4_, we observed odds ratios for the association of current contraceptive use with husbands’ positive perceptions of family planning that were consistently greater than 1. Within group_1_, the most significant odds ratios were found for husbands deeming family planning very important for spacing births, limiting the number of children, economic reasons and children’s health. Within group_4_, 6 out of the 7 enquired aspects of husband’s perception showed significance levels inferior to 0.05. The odds ratios for group_2_, on the other hand, were consistently inferior to 1 ranging from 0.24 to 0.78. For group_3_ no statistically significant odds ratio was found. Finally, as far as women’s perceptions are concerned, all odds ratios were greater than 1, without major differences across the groups (see Table 4).

Husband involvement, in particular his willingness to finance and purchase contraception, was almost consistently linked to higher contraceptive use, with odds ratios greater than 1. It was however of less significant importance in group_2_. A very high association was found between current contraceptive use and having discussed family planning within the couple in the past. Other variables related to decision-making practices within the couples were generally speaking not statistically significantly related to current contraceptive use. One exception however was the fact that in group_3_, we observed an odds ratio of zero (means showed larger difference) for current contraceptive use and having husbands as the only final decision-maker on family planning issues.

Multivariate analysis was performed in this study to examine the link between several factors and contraceptive use. Accordingly, variables showing a significant contribution to contraceptive use are wife’s age, husband’s desire for more children, husband’s favourable attitude towards family planning and husband’s preparedness to accept his wife’s use of contraceptives without his consent. In regard to the wife’s age, the result of the multivariate model displays that the younger the age of a woman, the better the woman experienced contraceptives. (p < 0.001 OR = 0.95).

The second factor that was identified as an important predictor of contraceptive use, is the husband’s desire for more children. In the groups where the husband does not want more children but the wife does, there is a 40% likelihood to use contraceptives (p = 0.03). When a husband has a favourable attitude, the women is twice more probable to practice family planning (OR = 2.17, p < 0.001) (see Table 5).

Discussion about family planning is associated with contraceptive practice. Compared to husbands’, wives’ experience of spousal discussion about family planning were nearly three times more likely to be related with the use of contraception by these couples (husbands OR = 2.39 p < 0.001 and wives OR = 5.37 p < 0.001). Willingness for his wife to use contraceptives without his consent was statistically significant with contraceptive use (OR = 1.54 p = 0.05) (see Table 5).

**Table 5:** Multivariate analysis of factors associated with couples contraceptive practice, Jimma zone, Ethiopia, 2014

	**Full model**				**Best model**			
	**OR**	**(95% CI)**		**p-**	**OR**	**(95% CI)**		**p-value**
**Age (yrs)**								
Husband	1.01	0.99	1.04	0.35				
Wife	0.94	0.9	0.97	<0.001	0.95	0.92	0.98	<0.001
**Desire to have more children**								
Husband	0.64	0.4	1.02	0.06	0.6	0.38	0.94	0.03
Wife	0.9	0.57	1.41	0.64				
**Husband supports his wife to use FP**	2.07	1.29	3.63	0.01	2.17	1.37	3.79	<0.001
**FP was previously discussed within the couple**								
Reported by the husband	2.48	1.54	4.02	<0.001	2.39	1.5	3.82	<0.001
Reported by the wife	5.79	3.78	9.01	<0.001	5.37	3.56	8.21	<0.001
**Husband is responsible for final decision-making regarding FP**								
Reported by the husband	0.74	0.34	1.6	0.44				
Reported by the wife	0.69	0.42	1.13	0.14	0.7	0.44	1.13	0.15
**Wife can use FP method without husband’s consent**								
Reported by the husband	1.45	0.92	2.28	0.11	1.54	1.01	2.36	0.05
Reported by the wife	1.23	0.82	1.86	0.33				

## Discussion

In recent years, there has been a growing, global call to invest more in family planning in order to reach MDG5 by 2015. In this context, a lot of research has been conducted to identify the socio-cultural barriers to family planning, including the impact of spouses’ intentions and attitudes towards contraception. However, much of this research studied wives and husbands independently instead of considering them as a dyad. Our study used the latter approach and explored determinants of contraceptive use linked to couple dynamics. Three important conclusions can be made based on this analysis.

First, we identified high levels of discordance between husband and wife in reported fertility desires. This highlights the importance of collecting data from both husband and wife separately in order to have a good understanding of the couple’s fertility desires, rather than relying on the information provided by one of the spouses exclusively. Although this recommendation had already been made in the past, we believe too many studies still fail to address this point [16]. A direct consequence of this gap concerns the concept of unmet need for family planning. The currently used definition for this concept only takes the desires and needs of married women or women living in union into account, without considering their husbands or partners. If both spouses consistently agreed on fertility preferences, this would not cause any problem, but our results show this is not the case. Becker [4] already noted that the calculated unmet need for contraception is significantly different for husband and wife [4,17] but this has not yet resulted in a new paradigm of assessing this indicator.

Our second conclusion relates to the finding that the highest levels of contraception were found in couples where only the husband did not want more children. This suggests that men may have more decision making power regarding contraceptive use than their wives, even though a majority of couples stated that any decision regarding family planning should be taken by both spouses jointly. Similar results were found in a study conducted in Egypt which revealed that a couple’s use of contraception is mostly determined by the husband’s desire for more children [18]. Furthermore, couples where the husband had a favourable attitude towards family planning showed higher levels of contraceptive use. Previous research indeed indicated that a husband’s fertility intention has an influence on the wife’s contraceptive adoption [19,20]. Notwithstanding these findings, most of the current family planning programs mainly focus on women rather than men. We therefore believe that significant improvements may be expected if men would be involved in a more systematic way and if activities to increase husband’s awareness about family planning were included in all programs [19-21].

Finally, previous spousal discussion of family planning issues was positively associated with contraceptive use, even though spouses were not always consistent in reporting this. Again, this is not a new finding since spousal communication has been reported as a strong determinant of a couple’s contraceptive use in several previous studies [22,23]. However, the fact that we found this relationship consistently across the four groups makes the linkage even stronger. For this reason, encouraging communication between partners could be the best means to increase the use of contraception by couples.

### Study limitations

One potential limitation of this study is that couples in which the wife was pregnant were not included, which restricted us not to compute unmet need for family planning. The other limitation of couple level analysis could be not considering the unmarried couples or couples in union or cohabited. Additionally, our results could also suffer from social desirability bias and recall bias. The social desirability bias includes the personal reproductive health history of each respondent, which is highly affected by cultural values held at the study set-up, that is a typical rural setting of Ethiopia. The other psychometric issues, such as gender norms and socio-cultural factors, were not assessed in detail in this study. These variables were mostly collected using qualitative methods which enabled to perform triangulation with our quantitative findings. Nonetheless, effort was exerted to overcome all the challenges faced during the study.

## Conclusion

This report has explored the extent to which spouses concord or discord regarding fertility desire and how this affects a couple’s contraceptive use. As the differences observed between spouses in fertility desire were substantial, it is essential, in order to identify mechanisms for better family planning practices, to not only take into account wives’ responses, but to involve men’s viewpoints as well. This recommendation is based upon the finding that in those couples where the wife is the only one wishing to limit pregnancies, well-informed husbands tend to obstruct their spouses’ access to family planning. In addition, the husband’s desire for more children determines greatly women’s contraceptive use. Hence, it is imperative to involve the husband in family planning programs for couples’ contraceptive use, precisely because their role in decision making is significant. In the future, family planning information, education and communication (IEC), including about the side effects and age variation of married couples, should be delivered to obtain a favourable attitude towards family planning. Further research shall be recommended, preferably using qualitative data collection methods, to include the psychometric aspect of couples using contraceptives. This method may help to validate the data and obtain a more thorough understanding of the results.

## Competing interests

The authors declare that they have no competing interests.

## Authors’ contributions

TT conceived the study, data analysis and writing process. GC contributed to conceiving the study and participated in writing. MT participated in conceiving the study. OD contributed significantly to the data analysis and writing process. All authors read and approved the final manuscript.
